# Cdk2 and Cdk4 cooperatively control the expression of Cdc2

**DOI:** 10.1186/1747-1028-1-10

**Published:** 2006-06-06

**Authors:** Cyril Berthet, Philipp Kaldis

**Affiliations:** 1 National Cancer Institute, Mouse Cancer Genetics Program, NCI-Frederick, Bldg.560/22-56, 1050 Boyles Street, Frederick, MD 21702-1201, USA

## Abstract

Progression through the mammalian cell cycle is associated with the activity of four cyclin dependent kinases (Cdc2/Cdk1, Cdk2, Cdk4, and Cdk6). Knockout mouse models have provided insight into the interplay of these Cdks. Most of these models do not exhibit major cell cycle defects revealing redundancies, and suggesting that a single Cdk might be sufficient to drive the cell cycle, similar as in yeast. Recent work on *Cdk2/Cdk4 *double knockouts has indicated that these two Cdks are required to phosphorylate Rb during late embryogenesis. The lack of Rb phosphorylation is progressive and associated with reduced E2F-inducible gene expression. Cdk2 and Cdk4 share the essential function of coupling the G1/S transition with mitosis. However, proliferation in early embryogenesis appears to be independent of Cdk2 and Cdk4. We discuss these observations and propose molecular mechanisms that establish the requirement for Cdk2 and Cdk4 at the G1/S transition. We are considering that the balance between proliferation and differentiation is disturbed, which affects especially heart development and leads to embryonic lethality in *Cdk2*^-/-^*Cdk4*^-/- ^mutants. We also discuss the specific functions of Cdk4 and Cdk6, which ironically do not compensate for each other.

## Background

Cell cycle regulation plays an essential role in cellular homeostasis and contributes to determine the fate of cells. Most factors influencing the decision, whether to start a new round of division or not, act at the G1/S transition. Mitogenic factors induce expression of cyclin D and therefore stimulate the activities of Cdk4 and Cdk6. The activation of the cyclin D/Cdk complexes is the first step leading to cell cycle entry and is followed by several waves of cyclin expression (cyclin E, cyclin A, and cyclin B). Each family of cyclins binds to a specific Cdk, which is active at a specific phase of the cell cycle and contributes to the activation of the next cyclin/Cdk complex. Recent studies in different Cdk knockout mice have challenged this common model of mammalian cell cycle regulation. Single loss of Cdk2, Cdk4, or Cdk6 did not significantly affect cell proliferation in vivo or in vitro [1-4]. Among the most surprising observations was the normal cell proliferation in *Cdk2 *knockout mice, though Cdk2 was considered to be a unique kinase bound to cyclin E, regulating S phase initiation and progression. This perplexing observation has been quickly addressed by further in vivo analysis demonstrating that Cdc2, which was previously demonstrated to control G2/M, is also able to bind cyclin E and compensates for Cdk2 in S phase [5]. Similarly, inactivation of both *Cdk4 *and *Cdk6 *does not affect cell cycle initiation and progression, suggesting that Cdk2 compensates for the lack of cyclinD dependent kinases [2]. More strikingly, the combined loss of Cdk6 and Cdk2 has no impact on cell proliferation and *Cdk2*^-/-^*Cdk6*^-/- ^mice display similar phenotypes as *Cdk6 *or *Cdk2 *single knockout mice [2]. These results suggest that a single G1/S phase associated Cdk is sufficient to induce cell cycle entry and progression through M phase. Based on this new knowledge, the mammalian cell cycle might not be very different from the yeast cell cycle, which is controlled by a single Cdk (Cdc2 or Cdc28). Yet, the mammalian cell cycle differs from the yeast cell cycle regarding the Rb/E2F pathway, which is essential for G1/S control. The Rb protein cycles between hypo and hyper phosphorylated forms and genes required for DNA replication and mitosis are repressed when E2F transcription factors are bound to hypophosphorylated Rb. Rb is a major substrate for Cdks and upon its phosphorylation, E2F proteins are released, which acts as an on/off switch for entry into S phase. Until recently, major Rb phosphorylation defects have never been observed in any of the "Cdk" or "cyclin" knockout mice. In vitro, it was shown that Cdk4, Cdk6, and Cdk2 phosphorylate Rb at different sites (for review, see [6]), but in vivo, one of these Cdks could be sufficient to accomplish Rb phosphorylation. We will discuss this hypothesis in reference to recent observations made in *Cdk2*^-/-^*Cdk4*^-/- ^mice and provide new models of mammalian cell cycle regulation.

## Cdk2 and Cdk4 cooperate to couple the G1/S transition with mitosis

We recently generated *Cdk2*/*Cdk4 *double knockout (DKO) mice and for the first time we observed reduced Rb phosphorylation in vivo and in vitro [7]. The decrease of Rb phosphorylation is progressive and does not occur before E13.5 during embryonic development. Rb protein levels are similar in wild type and DKOs, but phosphorylation at Serine 780 is decreased at E14.5 and barely detectable at E16.5. As a likely consequence, all embryonic tissues tested display a significant lower proliferation rate at E14.5. However, we still observed a high rate of overall proliferation in most tissues (i.e. lung, liver), suggesting that some cell subpopulations might be more affected than others. To better understand the molecular mechanism, we analyzed mouse embryonic fibroblasts (MEFs) and were able to correlate the lack of Rb phosphorylation with impaired S phase entry and premature senescence. The primary cause of the proliferation defect is associated with Rb hypophosphorylation and decreased expression of E2F-inducible genes, among them *Cdc2 *and *cyclin A2*. On the other hand, HPV-E7-mediated inactivation of Rb restored normal expression of E2F-inducible genes and cell proliferation. This result suggests that Cdk6 and Cdc2 can regulate cell proliferation, but these two kinases might not phosphorylate Rb to full extent, leading to decreased Cdc2 expression. The declining Cdc2 expression acts as a negative loop leading to proliferation defects. The fact that more Cdk6 is bound to cyclin D1, in the absence of Cdk4, is apparently not sufficient to compensate the lack of Cdk2 and Cdk4 (see below). From our experiments, we conclude that Cdk2 or Cdk4 is required, at a certain point, to phosphorylate Rb thereby maintaining higher levels of Cdc2 protein expression. These two G1/S kinases contribute to the activation of G2/M cyclin/Cdk complexes, and doing so, couple the G1/S transition with mitosis. Characterization of the double knockout mice of *Cdk2 *and *Cdk4 *revise the picture of the cell cycle, combining features from the classic mammalian model and features from the yeast model. Nevertheless, though we have uncovered the dynamics of this molecular mechanism, we still need to understand why Rb phosphorylation starts to decline only at midgestation.

## Why Cdc2 does not compensate for Cdk2 in late embryogenesis?

**Figure 1:**
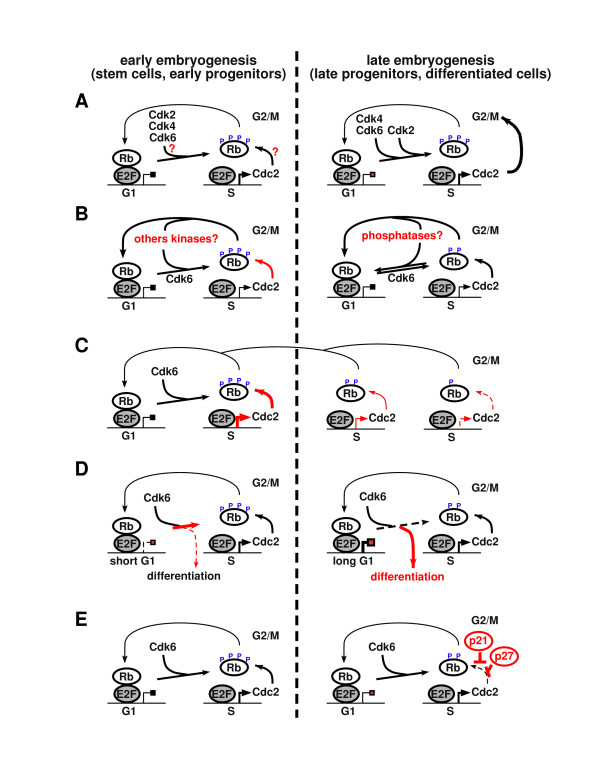
**Coupling of G1/S transition with mitosis**. Cell proliferation appears to be dependent on Cdk2 and Cdk4 in late embryogenesis but not in early embryogenesis. (A) Previous results suggested that phosphorylation of Rb is differently regulated in stem cells and in differentiated cells [10, 11]. The characterization of *Cdk2*^-/-^*Cdk4*^-/- ^mice demonstrates Cdk2 and Cdk4 independence for Rb phosphorylation in early embryogenesis and we proposed four models for the G1/S transition and the establishment of Cdk2/Cdk4 dependent Rb/E2F checkpoint. These molecular mechanisms are not exclusive and they might all play a role throughout the differentiation process or in specific cell types. (B) Specific kinases or phosphatases are differentially expressed in stem cells or differentiated cells, which modifies the phosphorylation of Rb. Cdc2 could be also more active in stem cells. (C) Cdk6 and Cdc2 do not phosphorylate Rb to full extend, which progressively affects the E2F-dependent transcription of Cdc2 and initiates a negative feedback loop. (D) Proteins promoting differentiation at the onset of the G1phase. Throughout the differentiation process, the length of G1phase extends, tipping the balance between proliferation and differentiation towards cell cycle exit and further differentiation. Unphosphorylated Rb itself is a major inducer of the cell cycle exit. (E) Increased expression of Cdk inhibitors in differentiated cells will affect Cdk activity (see text for discussion about *Cdk2*^-/-^*Cdk4*^-/-^*p27*^-/- ^cells).

The late embryonic lethality of DKOs suggest that Cdk2 and Cdk4 are required to regulate the Rb/E2F pathway only late in development. In agreement with this, cell proliferation in early embryogenesis is comparable in DKO and wild type embryos. Our analysis has shown that Rb phosphorylation decreases progressively after E13.5, and we can only speculate how Rb phosphorylation is maintained in early development. Therefore, we propose four possible models that are not mutually exclusive and can possibly explain our findings (Figure 1). The first possibility (Figure 1B) could be that in early embryogenesis, stem cell and early progenitor cell proliferation is not controlled by Rb and other pocket proteins (p107, p130). It has been shown that proliferation of embryonic stem (ES) cells is not affected by concomitant ablation of all three pocket proteins, whereas similar mutant MEFs proliferate faster than wild type MEFs and loose G1 control [8,9]. On the other hand, Rb appears to be immediately phosphorylated, and therefore inactivated, after completion of mitosis in ES cells, while the unphosphorylated form is observed at the onset of G1 phase in MEFs [10]. Moreover, cyclin D/Cdk4 associated kinase activity is undetectable and low levels of cyclin E/Cdk2 complexes are expressed in ES cells, consistent with an apparent lack of Rb regulation in ES cells [11]. As previously suspected, we can envision that in ES cells, there is a specific mechanism that triggers the inactivation of Rb and other pocket proteins. It could be a specific kinase, the absence of a phosphatase, the degradation of the pocket proteins, or simply an increased activity of Cdc2 (or Cdk6) in ES cells. This mechanism could be maintained through the early stages of embryogenesis and consequently the lack of Cdk2 and Cdk4 would have no effect until pocket proteins become active and repress E2F proteins. Our second model (Figure 1C) is based on exponential dynamics: at each cell division, the combined activities of Cdk6 and Cdc2 phosphorylate Rb but are not 100% efficient. The lack of efficiency might not be noticeable in early stages of embryogenesis, but after a number of cell divisions, Rb phosphorylation might fall below a threshold level, required to maintain high levels of Cdc2 expression. Consequently, low Cdc2 expression might affect noticeably the phosphorylation of Rb in the next round of cell division, thereby triggering the amplification of the negative loop. Our third model (Figure 1D) is based on the difference in G1 phase length between stem cells and differentiated cells. Indeed, the cell cycle length of stem cell is about 8 hours long, where as the differentiated cells take about of 20–24 hours to complete one cycle. This difference is mainly associated with a longer G1 phase that increases throughout the differentiation process. It is possible that Cdk6 and Cdc2 can completely phosphorylate Rb when G1 phase is short, but when the length of this phase extends their efficiency may drop. In this model, Cdc2 might not be active during early G1 to contribute with Cdk6 to the phosphorylation of Rb. Rb represses E2F and therefore S phase gene transcription during G1 phase, but on the other hand activates transcription in concert with other transcription factors (i.e. MyoD in myogenesis – for review, see [12]), and contributes to cell differentiation and cell cycle exit [13]. If Cdk activity is not high enough throughout the G1 phase, this balance might be shifted in favor of differentiation rather than proliferation. With an extended G1 phase, Cdk6 and Cdc2 might not be able to counteract the proteins inducing differentiation, even in the presence of growth factors. A fourth option (Figure 1E) is associated with the inhibitors of Cdks, p21 and p27. The combined loss of Cdk2 and Cdk4 most likely affects the distribution of these inhibitors since in wild type cells the majority of p27 is bound to Cdk4. Indeed, the inactivation of *Cdk4 *or *Cdk2 *induces relocation of free p27 to Cdk2 or Cdc2 complexes respectively, which affects their activity. Studies with *p27 *knockout mice have shown that loss of p27 can rescue a normal entry of S phase in *Cdk4*-null [4] or *Cdk2*-null MEFs (unpublished data). In *Cdk2*^-/-^*Cdk4*^-/- ^DKO, these inhibitors might be relocated to Cdc2 complexes and therefore, through their inhibition, contribute to the lack of Rb phosphorylation. We tested this hypothesis by knocking out p27 in *Cdk2*^-/-^*Cdk4*^-/- ^mice, but did not observe a rescue of embryonic lethality or proliferation of MEFs [7]. This result suggests that Cdk inhibitors, at least p27, are not responsible for Rb hypophosphorylation and proliferation defects in DKOs. To discriminate between these models, further studies will be necessary. Such studies could be done using stem cells, where proliferation and differentiation can be monitored, or by identification of molecular pathways that can rescue the lack of Rb phosphorylation. To address this, we have tried to overexpress Cdc2 in *Cdk2*^-/-^*Cdk4*^-/- ^MEFs but Cdc2 did not restore normal proliferation (unpublished data).

## Origin of *Cdk2*^-/-^*Cdk4*^-/- ^embryonic lethality

The embryonic lethality in DKOs is most likely associated with cardiac failure. The small size of the *Cdk2*^-/-^*Cdk4*^-/- ^embryos is probably linked to the progressive loss of Rb phosphorylation observed at midgestation. So far, we do not know if the heart defect is related to hypophosphorylation of Rb. A similar cardiac phenotype was observed in *cyclin D1-/-D2-/-D3-/- *triple knockouts [14]. It is likely that a common molecular mechanism affects the heart defects in *cyclin D*-null and *Cdk2*^-/-^*Cdk4*^-/- ^mice. *Cyclin D*-null mice do not display an Rb defect, however, it cannot be excluded that the Rb/E2F pathway is deregulated in *cyclin D*-null cardiomyocytes. This pathway plays a major role in cardiogenesis and the levels of free activated E2F is critical for normal cardiac function [15]. Another example of the strong Rb/E2F dependence in heart development is that hypophosphorylated Rb binds to a transcriptional repressor of cardiac specific genes, Jumonji [16]. The inactivation of Jumonji affects embryonic heart development and, in vitro, results in upregulation of cyclin D1 and Cdc2 levels, and increased cardiomyocyte proliferation [16]. From E11.5 to E14.5, cardiomyocytes proliferate rapidly, leading to expansion of the ventricular wall. In the heart of *Cdk2*^-/-^*Cdk4*^-/- ^mutants, Rb represses E2Fs, probably interacting with repressors like Jumonji, which then inhibits cardiomyocyte growth. Further characterization of this pathway might help to better understand the complexity of heart development and the relation to the cell cycle players. Studies of *Cdk2*^-/-^*Cdk4*^-/- ^cardiomyocytes will be a good experimental model to determine how the combined loss of Cdk2 and Cdk4 affects the balance between differentiation and proliferation. We need to determine why the cardiogenesis is more sensitive to inactivation of *Cdk2 *and *Cdk4 *than differentiation of other cell types.

## Cdk4 and Cdk6: similar kinases but not twins

Studies with double knockout mouse models have pointed out some differences between Cdk4 and Cdk6. Indeed *Cdk2*^-/-^*Cdk4*^-/- ^mutants are embryonic lethal, whereas *Cdk2*^-/-^*Cdk6*^-/- ^mice develop normally. Focusing on animal growth and control of cell proliferation, several observations suggest that Cdk4 and Cdk6 do not completely compensate for each other in vivo. *Cdk4 *single knockout males and females display reduced animal size [3,4], while inactivation of *Cdk6 *reduces the size of the females but to a lesser extend than *Cdk4 *mutation [2]. At the cellular level, S phase entry is delayed in *Cdk4*-null but not in *Cdk6*-null MEFs [2-4]. Moreover and in contrast to Cdk6, Cdk4 is able to promote a normal S phase entry in MEFs, in the absence of the other G1/S Cdks. This difference in the cell cycle regulation could be related to the reduced size of *Cdk4*^-/- ^and *Cdk2*^-/-^*Cdk4*^-/- ^mutants compared to wild type or *Cdk2*^-/-^*Cdk6*^-/- ^mutants. The complete inactivation of *Cdk4 *and *Cdk2 *leads to more pronounced lack of proliferation (at least in the hematopoietic linage and in MEFs) and affects cardiac development, thereby inducing embryonic lethality [7].

At the biochemical level, few differences have been described between Cdk4 and Cdk6 (for review, see [17]). In vivo, subtle differences in timing or pattern of expression could explain the divergence in phenotype of corresponding null animals (i.e. Cdk4 affects β-islet pancreatic cells, Cdk6 is involved lymphocyte T proliferation [2,3]). However, Cdk4 and Cdk6 are widely expressed in embryos and their expression is similar in most of the cell types, suggesting that these two kinases might be distinct regarding their substrate specificity. It has been shown that Cdk4 is more efficient in phosphorylating Rb than Cdk6 and displays different residue selectivity [18]. This result needs to be confirmed in vivo and could explain our observations in *Cdk2*^-/-^*Cdk4*^-/- ^mice. Other substrates might also be involved, such as Smad3, phosphorylated by Cdk4 and Cdk2 but not yet tested for Cdk6 [19]. Smad3 mediates growth inhibitory effects of TGFβ by upregulating the expression of Cdk inhibitors. Cdk4 and Cdk2 phosphorylate Smad3 and inhibit Smad3 antiproliferative function, providing negative feedback control. The lack of Cdk4 and Cdk2 might amplify this antiproliferative signal. Such crosstalk between cell cycle regulation and upstream signaling could affect the G1/S transition differently through Cdk4 or Cdk6. Moreover, recent findings suggest a new role for Cdk6 in the differentiation of a variety of cell types. This function, which affects the transcription of genes involved in terminal differentiation, is not shared with Cdk4 and could be independent of Rb (for review, see [17]). All these evidences gathered recently suggest that Cdk4 and Cdk6 may have independent functions in the maintenance of the delicate balance between cellular division and differentiation.

## Conclusion

Our knowledge of cell cycle regulation has greatly improved through the characterization of knockout mouse models. The overlap of Cdk functions adds more complexity to the in vitro model of the cell cycle (specific Cdk/cyclin complexes for each cell cycle phase). On the other hand, we can consider that all Cdks are redundant, which would result in a model similar to the yeast cell cycle. Our recent results show that the redundancy is not complete and each Cdk might have its own niche. This specificity could be essential for small subpopulations of cells (β-islet pancreatic cells for Cdk4, spermatocytes for Cdk2...) or affect cell cycle regulation globally. Indeed, Cdk2 and Cdk4 share a common role in the G1/S transition, which couples this phase with mitosis through E2F-inducible gene expression. Embryonic stem cells might proliferate independently of this coupling, and we presented four models to describe how this coupling can take place during embryogenesis. Among E2F-inducible genes, we focused on the role that Cdc2 can play as a kinase to phosphorylate Rb, however we cannot exclude that other E2F-targets are also important. Moreover, these four molecular mechanisms act probably in concert to establish the G1/S checkpoint. This role might be important with regards to cancer cells. Could the combined targeting of Cdk2 and Cdk4 be a valuable approach for cancer therapy? To answer this question, we have to determine if Rb wild type cancer cells require Cdk2 and Cdk4 activities throughout tumor progression. Future experiments with the *Cdk2*^-/-^*Cdk4*^-/- ^mouse model will teach us more details about tumorigenesis.

## Abbreviations

Cdk: cyclin dependent kinase

DKO: double knockout

ES cell: embryonic stem cells

MEF: mouse embryonic fibroblast

## Competing interests

PK is co-Editor-in-Chief of Cell Division but was not involved in the editorial and peer review process of this manuscript.
